# MicroRNA-429 Inhibits Microglial Inflammation by Targeting *IKKβ* Through the NF-κB Pathway

**DOI:** 10.1155/mi/7165782

**Published:** 2025-07-24

**Authors:** Zhongling Ke, Yanhui Chen, Xiaoxia Lin, Jilong Jiang

**Affiliations:** Fujian Medical University Union Hospital, Fuzhou, China

**Keywords:** inflammation, microglia, microRNA-429, Tourette syndrome

## Abstract

This study was designed to explore the specific mechanism of miR-429 in neuroinflammation of TS, to provide a theoretical basis for the etiology of TS and new targets for future treatment. Male SD rats were randomly divided into the normal control (CON) group and the Tourette syndrome (TS) group. The activation of microglia in the striatum was detected by immunohistochemistry and the concentration of interleukin (IL)-6 in the brain was measured by ELISA. The expression of miR-429 in brains was detected by quantitative real-time PCR (qPCR). Human microglia clone 3 (HMC3) cells were transfected, respectively, with double-stranded inactive miRNA; miR-429 mimics; miR-429 inhibitors. The levels of miR-429 and *IKKβ* mRNA were detected by qPCR. The expression levels of IKKβ, NF-κBp65 b, and IL-6 proteins were detected by western blot. MiR-429 and *IKKβ* targeted binding was verified by a double luciferase experiment. IL-6 in the brain of the TS rat group was higher than in the CON group. Furthermore, the relative expression of miR-429 in the TS group was several hundreds of times higher than in the CON group. The levels of IKKβ, NF-κBp65, and IL-6 protein in lipopolysaccharide (LPS)-induced microglia were lower in the miR-429 mimics group, but higher in the miR-429 inhibitor group, compared to those of the MiRNA negative control (miR-NC) group (*p* < 0.05). The miR-429 targeted *IKKβ* binding to regulate the NF-κB pathway and inhibit the release of pro-inflammatory factors, thus, controlling neuroinflammation in microglia, which may be the mechanism of action of miR-429 in TS.

## 1. Introduction

Tic disorders (TDs) are a group of neurological disorders that start in childhood and are characterized by multiple tics, motor, and/or vocal. The most severe type of TD is Tourette syndrome (TS), which can cause a wide range of psychosocial, physical, and functional difficulties that can make social activities, academic success, and career achievement difficult [1]. Although the cause and progression of TDs has not been fully understood, it is generally agreed that both genetic and environmental factors are likely responsible [2, 3]. The neurobiological mechanisms associated with TDs are still not completely understood. However, experts believe that they may involve abnormal cortico–striato–thalamo–cortical circuits (CSTCCs) and neurotransmitter imbalance in the CSTCC pathway [4].

In recent years, the hypotheses of the immunopathogenesis of TS has been increasingly recognized by scholars [5, 6]. There is growing evidence of neuroinflammatory factors in patients with TS. Wenzel et al. [7] found positive cerebrospinal fluid (CSF) oligoclonal bands in eight of 21 patients with TS of different ages. Baumgaertel et al. [8] found positive CSF oligoclonal bands in four of 20 adult patients with TS, with a positivity rate of 20%, which is higher than the 5% positivity rate in the normal population. And another study found a significant increase in the gene expression of interleukin (IL)-2, IL-2β receptors, and monocyte chemoattractant protein-1 (MCP-1) in the basal ganglia of TS patients [9]. All these studies suggested that neuroinflammatory may play an important role in the onset and development of TS.

MicroRNAs (miRNAs) are short RNA molecules of 19–25 nucleotides that regulate the posttranscriptional expression of target genes. A single miRNA can target and regulate hundreds of mRNAs and affect the expression of many genes involved in functional interaction pathways [10]. miRNAs may promote or limit inflammatory signaling to exacerbate or ameliorate the pathological consequences of neuroinflammation; the role of miRNAs in neuroinflammation makes them involved in the pathogenesis of several neurological diseases, such as autism, Parkinson's, and Alzheimer's disease [11]. Rizzo et al. [12] found significantly lower expression of miR-429 in Caucasian patients with TS than in normal controls and concluded that reduced serum levels of miRNA-429 were sufficient to distinguish TS patients from non-TS patients, with a sensitivity and specificity of 95% and 42%, respectively, and may serve as a biological marker for TS. However, the exact mechanism of miR-429 action is unclear.

MiR-429 was able to enhance NF-κB activity by downregulating the expression of *IKKβ* mRNA, which then increased the release of inflammatory factors in cervical cancer cells [13]. Thus, the experiments reported here were designed to assess whether miRNA-429 can also target binding *IKKβ* mRNA to regulate the NF-κB pathway to inhibit neuroinflammation in TS. While previous studies have linked neuroinflammation to TS, the role of miR-429 in modulating these pathways has not been fully explored. This study aims to address this gap by investigating the potential of miR-429 to regulate the NF-κB pathway and its implications for the treatment of TS.

## 2. Materials and Methods

### 2.1. Experimental Animal Modeling

A total of 20 21-day-old postnatal Sprague-Dawley (SD) male rats (weighing 60–70 g) were acquired from the Fuzhou Wu Lab Animal Center and housed in different cages at constant temperature and constant moisture (temperature level: 22 ± 2°C and moisture: 50% ± 10%), as well as fed and also watered freely. The rats were randomly split into the normal control (CON) group (*n* = 10) and the TS model group (*n* = 10). Rats in the CON group received an intraperitoneal (ip) injection of regular saline (1 mL/kg/day). The TS model was developed by ip injection of IDPN (TCI Shanghai Co., Ltd, Shanghai, China) at a dose of 150 mg/(kg/day) in a volume of 1 mL/kg for 7 successive days.

### 2.2. Collection of Samples

After behavioral evaluation, rats were weighed and anesthetized by a physician.

ip injection of 2% pentobarbital sodium (0.2 mL/100 g) and placed on the operating table in a supine position. The abdominal cavity, diaphragm, and chest cavity were subsequently opened. The heart was then quickly flushed with 100 mL of regular saline until the effluent was transparent, then, the rats were beheaded and the brains were rapidly dissected. The left hemisphere was immersed in 4% paraformaldehyde for fixation and subsequently processed for immunohistochemical analysis of microglial activation. The frontal cortex, striatum, and thalamus were dissected from the right hemisphere and stored at −80°C for subsequent ELISA and quantitative real-time PCR (qPCR) analyses.

### 2.3. Cell Culture

Human microglia clone 3 (HMC3) cells and human embryonic kidney-293T (HEK-293T) cells were purchased from Procell Life Science & Technology Co., Ltd (Wuhan, China). HMC3 were cultured in Dulbecco's modified eagle medium (DMEM; Procell Life Science & Technology Co., Ltd, Wuhan, China) with low glucose, 10% fetal bovine serum (FBS); and HEK-293T cells were cultured in DMEM (Procell Life Science & Technology Co., Ltd, Wuhan, China) with high glucose, 10% FBS. The cells were maintained in a 37°C sterile humidified incubator containing 5% CO_2_.

### 2.4. Transfection

MiRNA negative control (miR-NC) miR-429 mimics, miR-429 inhibitor, were purchased from GenePharma Co., Ltd (Shanghai, China). HMC3 cells were seeded in 6-well plates (2 × 10^5^/well), cultured at 37°C, with 5% CO_2_ for 16 h. Cells were transfected with DNAfectinTM Plus (Applied Biological Materials, Co., Ltd, Zhenjiang, China) containing miR-NC, miR-429 mimics, and miR-429 inhibitors according to the supplier's instructions.

### 2.5. Lipopolysaccharide (LPS) Intervention

At 24 h after transfection, the cell was exposed to LPS (100 ng/mL) to induce microglial inflammation.

### 2.6. Western Blot Analysis

Total cell proteins were extracted using RIPA buffer. The protein concentration was measured by the BCA assay. Protein samples were separated by electrophoresis using 10% separation gel and then the protein was transferred for 85 min to the PVDF membrane. The membrane was blocked with 5% skim milk for 2 h at room temperature and rinsed three times with TBST for 10 min each. Primary antibodies, rabbit anti-IKKβ (1:1000; Beyotime Co., Ltd, Shanghai, China), rabbit anti-Phospho-IKKα/β (1:1000; Beyotime Co., Ltd, Shanghai, China), rabbit anti-NF-κBp65 (1:1000; Beyotime Co., Ltd, Shanghai, China), mouse anti-IL-6 (1:1000; proteintech Co., Ltd, Wuhan, China), and mouse anti-β-actin (1:1000; Proteintech Co., Ltd, Wuhan, China) were incubated with the membrane at 4°C overnight. The membrane was then rinsed three times with TBST. Subsequently, goat anti-rabbit IgG (HRP; 1:10,000; Beyotime Co., Ltd, Shanghai, China) and goat anti-mouse IgG (HRP; 1:10,000; Proteintech Co., Ltd, Wuhan, China) were added and incubated at room temperature for 1 h. The membrane was rinsed three times with TBST for 10 min each, immunoreactivity was detected by ECL. Image J software was used for quantitative analysis.

### 2.7. ELISA

IL-6 levels in the rat hemicerebrum were detected with ELISA kits (Boster Biological Technology, Co., Ltd, Wuhan, China), according to the manufacturer's instructions. Each sample was analyzed in duplicate to ensure consistency and accuracy in the measurements.

### 2.8. RNA Isolation and Reverse Transcription (RT)-PCR

qRT-PCR was used to determine the expression of miR-429 and *IKKβ*. Each sample was analyzed in triplicate to ensure consistency and reliability of the results. RNA was extracted and the reverse transcription reaction was carried out according to the instructions of the HieffmiRNA 1st Stand cDNA Synthesis Kit (Yeasen Biotech Co., Ltd, Shanghai, China) for miR-429 and the reverse transcription reaction was carried out according to the instructions of the 5× All-In-One RT MasterMix kit (Applied Biological Materials, Co., Ltd, Zhenjiang, China) for *IKKβ*, using a 20 μL system for 30 cycles. The Hieff miRNA miRNA Universal qPCR SYBR Master kit (Yeasen Biotech Co., Ltd, Shanghai, China) for miR-429 and the SYBR Green qPCR Master Mix kit (Yeasen Biotech Co., Ltd, Shanghai, China) for *IKKβ* was used for PCR, all PCR were performed on the ABI7500 system. GADPH or U6 was an internal reference. The primer sequences were as follows:

**Table:** 

RNA	Forward primer	Reverse primer
U6	5′-CTCGCTTCGGCAGCACA-3′	5′-AACGCTTCACGAATTTGCGT-3′
miR-429	5′-CCGCGTAATACTGTCTGGTAAAACCGT-3′	Universal 3′ miRNA reverse primer (Yeasen Biotech Co., Ltd Shanghai, China)
*GAPDH*	5′-GGTGTGAACCATGAGAAGTATGA-3′	5′-GAGTCCTTCCACGATACCAAAG-3′
*IKKβ*	5′-GGCTGAAGCACATAACCTCTG-3′	5′-GCTCTTCTTCCGTCTGTAAC-3′

### 2.9. Dual-Luciferase Assay

The potential binding site between miR-429 and *IKKβ* was predicted by DIANA Software. 3′-UTR of *IKKβ* wild-type (WT) and mutant sequences connected to the double luciferase reporter gene vector (vector plasmid: pmirGLO, cloning site: Sacl-Sall) were synthesized by Shangya Biotechnology Co., Ltd (Zhejiang, China) and then, HEK-293T cells were cotransfected with pmirGLO luciferase reporter and miR-429 mimics or miR-NC. 48 h after transfection, luciferase activities were determined using a dual luciferase reporter assay system (Promega Co., Ltd, Beijing, China).

### 2.10. Statistical Analysis

The experimental data were analyzed by R software. Statistical graphs were also drawn using R software. Data were revealed as mean ± standard error (mean ± SEM). All experiments were carried out three to five times. One-way analysis of variance (ANOVA) was applied to compare more than one independent constant variable with normal distribution data followed by post hoc analyses with the Tukey HSD test, while independent and nonnormally distributed constant variable data were analyzed using the Kruskal Wallis test followed by post hoc analyzes with the Steel–Dwass test and the correlation relationships were analyzed by Pearson correlation analysis. *p* < 0.05 was considered statistically significant.

## 3. Results

### 3.1. The Level of IL-6 in TS Brain Tissue

**Figure 1:**
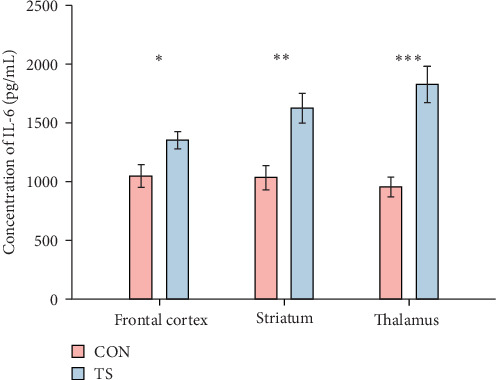
Comparison of the level of IL-6 between the TS and CON groups. The IL-6 level in different area (frontal cortex, striatum, and thalamus) of the brain in the TS group was higher than in the CON group. *⁣*^*∗*^*p* < 0.05; *⁣*^*∗∗*^*p* < 0.01; *⁣*^*∗∗∗*^*p* < 0.001.

The level of IL-6 in brain tissue from the TS group was significantly higher than that of the CON group (*p* < 0.05; Table 1 and Figure 1).

### 3.2. miR-429 Was High in TS

**Figure 2:**
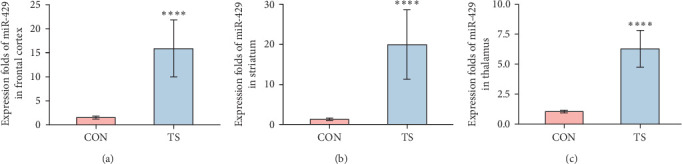
Comparison of miR-429 expression in brain tissues of rats of the TS and CON group. The expression of miR-429 in the brain of rats was higher in the TS group than in the CON group. (A) Expression folds of miR-429 in frontal cortex. (B) Expression folds of miR-429 in striatum. (C) Expression folds of miR-429 in thalamus. CON, normal control; TS, Tourette syndrome. *⁣*^*∗∗∗∗*^*p* < 0.0001.

The relative expression level of miR-429 in the TS group was higher than that of the CON group and the difference was statistically significant (*p* < 0.01; Table 2 and Figure 2).

### 3.3. Correlation Analysis of IL-6 Level and miR-429 Relative Quantification

**Figure 3:**
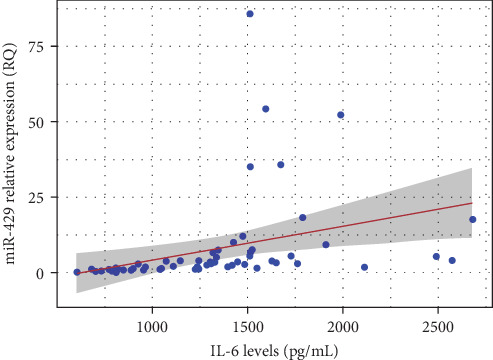
Correlation between IL-6 concentration and relative quantification (RQ) of miR-429 in brain tissue. A moderate positive correlation was observed (Pearson's *r* = 0.348, *p*=0.006). The red line represents the linear regression fit and the shaded area indicates the 95% confidence interval.

Pearson's correlation analysis revealed a moderate positive correlation between IL-6 concentration and the relative quantification (RQ) of miR-429 in brain tissue (*r* = 0.348, *p*=0.006). This association was statistically significant, with a 95% confidence interval ranging from 0.103 to 0.553, suggesting that higher IL-6 levels are associated with increased miR-429 expression (Figure 3).

### 3.4. Comparison of Microglial Activation Between the TS Group and Normal Controls

**Figure 4:**
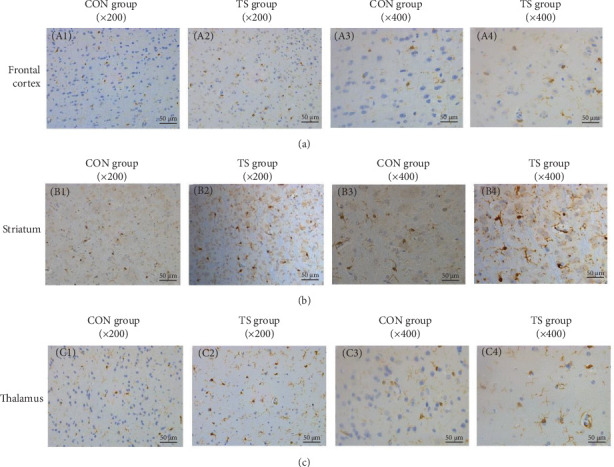
Immunohistochemistry of microglia in TS group and normal control group. CON, normal control; TS, Tourette syndrome. (A1)–(A4)(A) Frontal cortex: (A1) normal control group (×200), (A2) TS group (×200), (A3) normal control group (×400), and (A4) TS group (×400). (B1)–(B4)(B) Striatal areas: (B1) normal control group (×200), (B2) TS group (×200), (B3) normal control group (×400), and (B4) TS group (×400). (C1)–(C4)(C) Thalamus: (C1) normal control group (×200), (C2) TS group (×200), (C3) normal control group (×400), and (C4) TS group (×400).

Immunohistochemical analysis showed that activated microglia in the TS group appeared as brown-stained cells with enlarged cell bodies and thickened and shortened processes (Figure 4). Quantitative analysis revealed that the average optical density (AOD) values of microglia in the frontal cortex, striatum, and thalamus were significantly higher in the TD group than in the CON group (*p* < 0.001), indicating enhanced microglial activation in these brain regions (Table 3).

### 3.5. miR-429 Inhibits Human Microglia Inflammation Through the IKKβ/NF-κB Pathway In Vitro

#### 3.5.1. miR-429 Reduced LPS-Induced IL-6 Production by IKK*β*/NF-κB Pathway in Microglia

**Figure 5:**
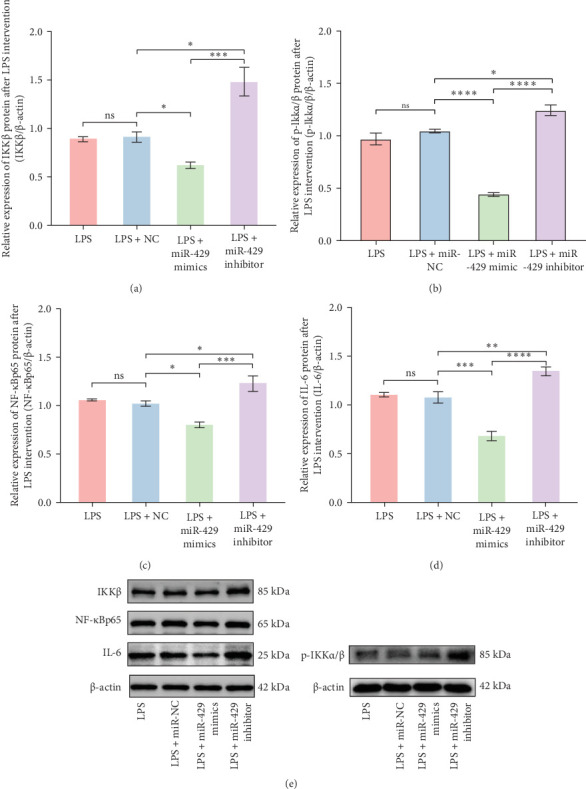
The differential expression of LPS-induced inflammatory protein in microglia transfected with miR-429 mimics or inhibitors. The expression of the LPS-induced inflammatory proteins IKK*β* (A), p-IKK*α*/*β* (B), NF-κB (C), and IL-6 (D) was lower in the miR-429 mimic group, but higher in the miR-429 inhibitor group, compared to the miR-NC group. The expression of the protein was examined by western blot (E). ns, no significant. *⁣*^*∗*^*p* < 0.05; *⁣*^*∗∗*^*p* < 0.01; *⁣*^*∗∗∗*^*p* < 0.001; *⁣*^*∗∗∗∗*^*p* < 0.0001.

To obtain further direct evidence that miR-429 is involved in the inflammatory response of microglia, we assessed the effects of overexpressing and inhibiting miR-429 on LPS-induced pro-inflammatory cytokine production (IL-6) in vitro. miR-429 mimics reduced the secretion of IL-6, whereas miR-429 inhibitor increased the production of IL-6. Similarly, miR-429 also reduced the secretion of IKKβ, Phospho-IKKα/β, and NF-κB. These results indicate that miR-429 attenuates the production of LPS-induced inflammation in microglia by IKKβ/NF-κB pathway (Table 4 and Figure 5).

#### 3.5.2. miR-429 Binds to IKK 3'-UTR′ of *IKKβ* and Inhibits IKK*β* Expression

**Figure 6:**
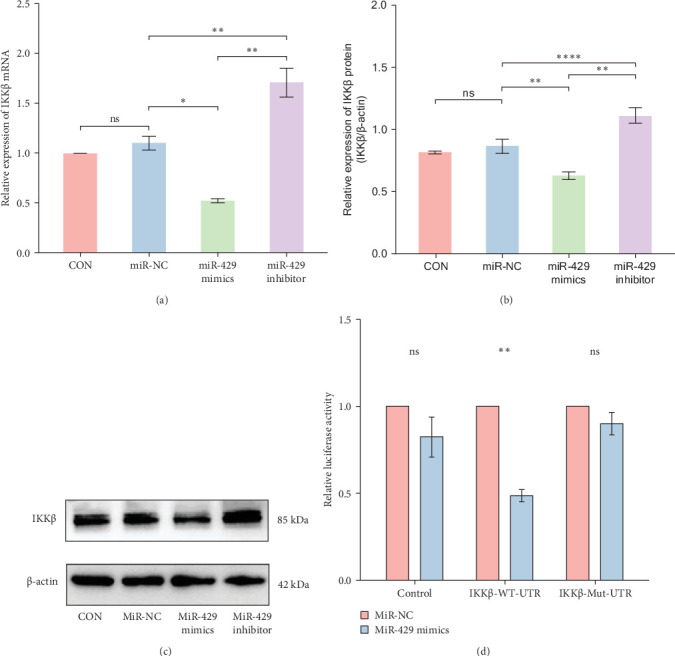
miR-429 is directly bound to *IKKβ* directly and inhibits its expression. (A) The expression of *IKKβ* mRNA decreased after transfected with miR-429. (B, C) The expression of IKKβ protein decreased after transfected with miR-429. (D) The results of the dual luciferase assay showed that miR-429 markedly inhibited the activity of luciferase for wild-type 3′-UTR of IKKβ. ns, no significant. *⁣*^*∗*^*p* < 0.01; *⁣*^*∗∗*^*p* < 0.01; *⁣*^*∗∗∗∗*^*p* < 0.0001.

Since miR-429 expression was significantly increased in TS rats and decreased in HMC3 after stimulation of LPS, we hypothesized that miR-429 played an important role in the regulation of microglial inflammatory responses. *IKKβ* was considered to be the target of miR-429 in oncological cells. To verify *IKKβ* as a target gene for miR-429 in human microglia, we constructed a low- and overexpressed miR-429 cell model with HMC3. We transfected HMC3 with miR-429 mimics, miR-429 inhibitor, and miR-NC, and then, examined the expression of *IKKβ*. As shown in Table 5 and Figure 6A, miR-429 transfection significantly inhibitor the *IKKβ* mRNA expression compared to miR-NC, while miR-429 inhibitor transfection significantly promotes the *IKKβ* mRNA expression. We then analyzed the effect of miR-429 on the protein level of IKKβ by western blot analysis. As shown in Table 6 and Figure 6B,C, transfection with miR-429 mimics significantly decreased the protein level of IKKβ compared to miR-NC, while miR-429 inhibitor transfection significantly promotes the IKK*β* protein expression. To further confirm that miR-429 was able to directly bind to IKKβ mRNA and then, inhibited the expression of IKKβ, we generated luciferase reporter constructs by cloning either the WT or a mutated portion of IKK 3′-UTR in the 3′-UTR of *IKKβ* into the 3′-UTR of a pmirGLO vector and transfected into HEK-293 cells. As shown in Table 5 and Figure 4D, we found that miR-429 transfection markedly inhibited luciferase activity for WT 3′-UTR of *IKKβ*, but showed no repression effect for mutated 3′-UTR of *IKKβ* and miR-429 and miR NC transfection had no significant effect on the empty pmirGLO vector. These results implied that miR-429 can bind to IKKβ directly and inhibit its expression.

## 4. Discussion

In this study, we found that the expression of IL-6 and miR-429 in brain tissue of the IDPN-constructed TS rat model was significantly higher than that of the CON group and miR-429 was able to target binding to *IKKβ*, affecting the NF-κB pathway and inhibiting the release of IL-6 inflammatory factors. These indicated that miR-429 played a vital role in neuroinflammation of TS and that miR-429 may serve as a biological marker and therapeutic target for TS in the future.

Our study found elevated levels of the cytokine IL-6 in the rat brain of the TS group compared to the control group. IL-6 was thought to be a associated factor for many neuropsychiatric diseases [14], such as Alzheimer's disease, [15] and depressive disorder [16]. It also had a close relationship with TD. Tao et al. [17] found that the serum IL-6 concentration of children with TD was significantly higher than that of the control group, which was consistent with other research [18, 19]. Cytokines promote cell communication with each other, coordinate complex multicellular behaviors and play an important mediating role between the immune system and the nervous system [20]. Chronic production of certain cytokines (e.g., IL-6) in the central nervous system is responsible for the development of human neurological disorders [21]. For ethical reasons, CSF examination is not necessary for TS, there was very limited research on CSF examination and no report of IL-6 concentration in patients with TS. However, abnormalities in peripheral immune function can impact the central nervous system's inflammatory response [22]. Therefore, increased IL-6 in peripheral blood may contribute to central nervous inflammation. And in the animal model of TS, we found an elevated level of IL-6 in the brains of TS rats, which was consistent with prior reports [23]. This suggested that IL-6 may play a key role in TS inflammation.

We also found that the expression of miR-429 in brain tissue of the TS rat model was much higher than that of the CON group, consistent with IL-6; suggesting that miR-429 may play an important role in inflammation. miR-429, a member of the miR-200 family, was found to bind to IKKβ to inhibit NF-κB release in oncology studies [24]. This is similar to our findings that miR-429 inhibited microglia inflammation by targeting the binding of *IKKβ* to regulate the NF-κB pathway. Microglia are innate immune cells of the central nervous system and play an important role in neuroinflammation [25]. Activated microglia could release many pro-inflammatory factors, such as IL-6, IL-1β, and TNF-α [26]. Microglia have been reported to play a role in TS neuroinflammation [27–29]. NF-κB was a major pathway in microglial activation [30, 31] and IKKβ was an important mediator in the regulation of the NF-κB signaling pathway [32, 33]. In our study, the dual luciferase assay implied that miR-429 can bind to *IKKβ* directly and further analysis showed that miR-429 could inhibit the expression of *IKKβ* mRNA and protein, leading to a reduction in phosphorylated IKKα/β protein expression, which indicated that miR-429 could influence the NF-κB single pathway.

Our subsequent experiments showed that human microglia transfected with miR-429 mimics had lower protein expression of IKKβ, phosphorylated *IKKα/β*, NF-κBp65, and IL-6, which were proteins related to NF-κB pathway, than the negative control group in LPS-induced inflammation, while in the group transfected with the miR-429 inhibitor, the expression of IKKβ, phosphorylated IKKα/β, NF-κBp65, and IL-6 was much higher than that of the negative control. These findings confirmed that in human microglia, miR-429 was able to target binding to *IKKβ*, affecting the NF-κB pathway, and then, inhibit the release of inflammatory factors such as IL-6. This may explain the pathogenic mechanism of miR-429 in TS, as follows: Low expression of serum miR-429 in patients with TS may not inhibit the activation of the NF-κB pathway after infection, which in turn leads to abnormal activation of microglia and then causes a neuroinflammatory response in TS.

However, our study has some limitations, such as being limited to animal and cellular experiments. If miR-429 and inflammatory factors in CSF can be collected in clinical patients in the future to clarify their correlation, this would further support our findings.

## 5. Conclusions

In conclusion, the results of the present study indicate that miR-429 can target *IKKβ* directly to regulate the activation of NF-κB and subsequently inhibit the inflammatory response of LPS-induced microglia. These results could lead to the identification of new potential therapeutic targets to control inflammation in TS.

## Figures and Tables

**Table 1:** Comparison of the level of IL-6 between the TS and CON groups ($\overset{-}{x}$ ± SEM, pg/mL).

Groups	Frontal cortex	Striatum	Thalamus
CON	1046.84 ± 317.93	1033.73 ± 323.4	956.39 ± 263.64
TS	1352.03 ± 232.92	1624.81 ± 395.63	1826.26 ± 482.8
*p*-Value	0.0259	0.0019	0.0002

Abbreviations: CON, normal control; TS, Tourette syndrome.

**Table 2:** Comparison of miR-429 expression (2^−*ΔΔ*CT^) in brain tissues of rats of the TS and CON group ($\overset{-}{x}$ ± SEM).

Groups	Frontal cortex	Striatum	Thalamus
CON	1.53 ± 0.32	1.3 ± 0.28	1.05 ± 0.11
TS	15.87 ± 5.88	19.95 ± 8.66	6.25 ± 1.53
*p*-Value	4.33e−05	4.33e−05	1.083e−05

*Note: ΔΔ*CT was calculated using the mean of *Δ*CT of normal controls as a control.

Abbreviations: CON, normal control; TS, Tourette syndrome.

*⁣* ^*∗*^ *p* < 0.05.

**Table 3:** Comparison of microglia activation in brain tissue between TS group and normal control group (AOD, $\overset{-}{x}$ ± SEM).

Groups	Fontal cortex	Sriatum	Talamus
CON	1.54 ± 0.09	1.35 ± 0.1	1.87 ± 0.13
TS	3.22 ± 0.11	2.62 ± 0.27	3.19 ± 0.19
*t*-Value	11.662	4.444	5.8973
*p*-Value	0.0000	0.0009	0.0000

Abbreviations: CON, normal control; TS, Tourette syndrome.

**Table 4:** LPS-induced production of inflammatory proteins in microglia after miRNA transfection.

Group	IKKβ/β-actin	p-IKKα/β/β-actin	NF-κB/β-actin	IL-6/β-actin
LPS	0.89 ± 0.02	0.97 ± 0.10	1.06 ± 0.01	1.10 ± 0.02
LPS + miR-NC	0.91 ± 0.05	1.04 ± 0.03	1.02 ± 0.03	1.07 ± 0.06
LPS + miR-429 mimics	0.62 ± 0.03	0.44 ± 0.03	0.8 ± 0.03	0.68 ± 0.05
LPS + miR-429 inhibitor	1.48 ± 0.15	1.24 ± 0.09	1.23 ± 0.08	1.34 ± 0.05
*p*-Value	≤0.001	≤0.001	≤0.001	≤0.001

Abbreviations: LPS, lipopolysaccharide; NC, negative control; p-IKKα/β, phospho-IKKα/β.

**Table 5:** The expression of *IKKβ* mRNA and protein after transfected with miR-429.

Group	*IKKβ* (mRNA)	IKKβ/β-actin (protein)
CON	1 ± 0	0.82 ± 0.01
miR-NC	1.1 ± 0.07	0.87 ± 0.06
miR-429 mimics	0.52 ± 0.02	0.63 ± 0.03
miR-429 inhibitor	1.71 ± 0.15	1.11 ± 0.06
*p*-Value	0.02	≤0.001

Abbreviations: CON, normal control; NC, negative control.

**Table 6:** Differences in the relative luciferase activity among groups in dual luciferase assay.

Group	Control	IKKβ-WT-UTR	IKKβ-Mut-UTR
miR-NC	1 ± 0	1 ± 0	1 ± 0
miR-429 mimics	0.82 ± 0.11	0.49 ± 0.04	0.90 ± 0.06
*p*-Value	0.2625	0.0048	0.2554

Abbreviations: Mut, mutated-type; NC, negative control; UTR, untranslated region; WT, wild-type.

## Data Availability

The datasets used and/or analyzed during the current study are available from the corresponding author upon reasonable request.
